# Effect of Epinephrine Administered during Cardiopulmonary Resuscitation on Cerebral Oxygenation after Restoration of Spontaneous Circulation in a Swine Model with a Clinically Relevant Duration of Untreated Cardiac Arrest

**DOI:** 10.3390/ijerph18115896

**Published:** 2021-05-31

**Authors:** Hyoung Youn Lee, Kamoljon Shamsiev, Najmiddin Mamadjonov, Yong Hun Jung, Kyung Woon Jeung, Jin Woong Kim, Tag Heo, Yong Il Min

**Affiliations:** 1 Trauma Center, Chonnam National University Hospital, 42 Jebong-ro, Dong-gu, Gwangju 61469, Korea; apostle09@naver.com; 2 Department of Medical Science, Chonnam National University Graduate School, 160 Baekseo-ro, Dong-gu, Gwangju 61469, Korea; shamsievkamoljon@gmail.com (K.S.); doctornm92@mail.ru (N.M.); 3 Department of Emergency Medicine, Chonnam National University Hospital, 42 Jebong-ro, Dong-gu, Gwangju 61469, Korea; xnxn77@hanmail.net (Y.H.J.); docheo@hanmail.net (T.H.); minyi46@hanmail.net (Y.I.M.); 4 Department of Emergency Medicine, Chonnam National University Medical School, 160 Baekseo-ro, Dong-gu, Gwangju 61469, Korea; 5 Department of Radiology, Chosun University Hospital, 365 Pilmun-daero, Dong-gu, Gwangju 61453, Korea; jw4249@hanmail.net

**Keywords:** heart arrest, epinephrine, oxygen, hypoxia

## Abstract

Severe neurological impairment was more prevalent in cardiac arrest survivors who were administered epinephrine than in those administered placebo in a randomized clinical trial; short-term reduction of brain tissue O_2_ tension (PbtO_2_) after epinephrine administration in swine following a short duration of untreated cardiac arrest has also been reported. We investigated the effects of epinephrine administered during cardiopulmonary resuscitation (CPR) on cerebral oxygenation after restoration of spontaneous circulation (ROSC) in a swine model with a clinically relevant duration of untreated cardiac arrest. After 7 min of ventricular fibrillation, 24 pigs randomly received either epinephrine or saline placebo during CPR. Parietal cortex measurements during 60-min post-resuscitation period showed that the area under the curve (AUC) for PbtO_2_ was smaller in the epinephrine group than in the placebo group during the initial 10-min period and subsequent 50-min period (both *p* < 0.05). The AUC for number of perfused cerebral capillaries was smaller in the epinephrine group during the initial 10-min period (*p* = 0.005), but not during the subsequent 50-min period. In conclusion, epinephrine administered during CPR reduced PbtO_2_ for longer than 10 min following ROSC in a swine model with a clinically relevant duration of untreated cardiac arrest.

## 1. Introduction

Since Crile and Dolley first described the use of epinephrine as a treatment for cardiac arrest in 1906 [1], it has been the first-line drug used during cardiopulmonary resuscitation (CPR). The administration of epinephrine during CPR is primarily intended to increase coronary perfusion pressure (CPP) and myocardial blood flow, thereby facilitating the restoration of spontaneous circulation (ROSC). Multiple clinical studies have suggested that epinephrine increases the rate of ROSC but fails to improve neurologically favorable survival, the ultimate goal of resuscitation [2,3]. Despite these results, current CPR guidelines still recommend the administration of epinephrine in cardiac arrest patients [4].

In the Prehospital Assessment of the Role of Adrenaline: Measuring the Effectiveness of Drug Administration in Cardiac Arrest (PARAMEDIC2) trial [3], the largest randomized double-blind clinical trial of epinephrine versus saline placebo for adult out-of-hospital cardiac arrest to date, patients in the epinephrine group had higher ROSC and survival rates at 30 days. However, these benefits of epinephrine did not translate into improved neurologically favorable survival because the epinephrine group contained significantly more survivors with severe neurological impairment than the placebo group. The authors of this study suggested that the adverse effects of epinephrine on cerebral oxygenation, which were reported in a study by Ristagno et al. [5], might be causally responsible for the higher rate of severe neurological impairment in the epinephrine group. Ristagno et al. investigated the effects of epinephrine administered during CPR on cerebral microcirculation and oxygenation in pigs that underwent 3 min of untreated ventricular fibrillation (VF) cardiac arrest followed by CPR [5]; the animals treated with epinephrine showed more pronounced reductions in cerebral cortical microcirculatory blood flow (MBF) and brain tissue O_2_ tension (PbtO_2_) immediately after ROSC than did the animals that received saline placebo. However, the MBF recovered to normal values and the difference in PbtO_2_ between epinephrine and placebo groups reversed within 10 min of ROSC, which resulted in a duration of exposure to PbtO_2_ levels below the commonly used hypoxic threshold (20 mmHg) of less than 2 min in the epinephrine group [6]. These short-lived adverse effects of epinephrine on cerebral oxygenation seem insufficient to explain the poorer neurological outcome among the epinephrine-treated survivors in the PARAMEDIC2 trial. The rapid recovery from cerebral hypoxia in the study by Ristagno et al. might have resulted from the short duration of untreated cardiac arrest in their study: the 3-min interval before treatment might not be sufficient to induce clinically relevant disturbances of cerebral oxygenation after cardiac arrest [7,8,9]. Thus, the effects of epinephrine administered during CPR on cerebral oxygenation after ROSC need to be determined in a more severe and clinically relevant cardiac arrest model in order to better understand its impact on subsequent cerebral injury.

In the present study, we investigated the effects of epinephrine administered during CPR on cerebral oxygenation after ROSC in a swine model with a clinically relevant duration of untreated cardiac arrest. We hypothesized that under these conditions, epinephrine treatment would result in significantly lower PbtO_2_ than saline placebo administration, during both the initial 10-min and subsequent 50-min post-ROSC periods.

## 2. Materials and Methods

This prospective experimental study was conducted in accordance with the National Institutes of Health Guide for the Care and Use of Laboratory Animals on 24 healthy Yorkshire/Landrace cross pigs weighing 24.5 ± 3.0 kg. The Animal Care and Use Committee of Chonnam National University Hospital approved this study (CNUH IACUC-21020). The investigators who undertook the experiments had completed an Animal Care and Use Committee training program. Sevoflurane anesthesia was used during all surgical interventions, and every effort was made to minimize the suffering of the animals.

### 2.1. Animal Preparation

Prior to the experiments, the animals were housed in a temperature- and light-controlled room (22 °C; 12-h light/dark cycle) for at least 7 days, with free access to tap water and commercial feed. After initial sedation with intramuscular ketamine (20 mg/kg) and xylazine (2.2 mg/kg) followed by a mixture of inhaled sevoflurane (2–5%) and O_2_ via a nose cone, each animal’s trachea was intubated with a cuffed endotracheal tube (6.5-mm internal diameter). Mechanical ventilation was achieved using an anesthesia machine on 70%:30% N_2_O:O_2_ and 1–2.5% sevoflurane with the following settings: tidal volume, 10 mL/kg; ventilatory rate, 12 breaths/min (subsequently adjusted to achieve normocapnia); inspiratory/expiratory ratio, 1:2; and positive end-expiratory pressure, 5 cmH_2_O. During the animal preparation procedures, the inhaled sevoflurane was titrated to maintain adequate anesthesia (absence of reflex withdrawal and no change in respiratory rate, heart rate, or arterial pressure). An end-tidal CO_2_ sampling line (B40 patient monitor; GE Healthcare, Chalfont St Giles, UK) was connected between the tracheal tube and ventilator circuit. A 7.0-F double-lumen catheter was advanced from the left femoral artery into the lower abdominal aorta for arterial blood sampling and pressure monitoring. A 7.0-F introducer sheath was advanced from the right external jugular vein into the right atrium for pacing catheter insertion and right atrial pressure monitoring. A 6.0-F single-lumen catheter was inserted into the left internal jugular vein and passed as far toward the base of the skull as possible for jugular venous blood sampling. After infiltration of 2% lidocaine solution into the scalp, a scalp incision was made to expose the right and left parietal regions of the skull. Cranial burr holes (10 mm in diameter) were drilled bilaterally over the right and left parietal cortices, and the dura mater underneath each burr hole was opened to expose the parietal cortex. Adhesive electrodes were applied to the limbs for electrocardiogram recording. Rectal temperature was maintained at 37.5 ± 0.5 °C using a heating blanket. An investigator, otherwise uninvolved with this study, assigned the animals to the placebo group or the epinephrine group according to the information in a closed envelope and prepared the syringes, each containing 10 mL of either saline placebo or epinephrine 20 μg/kg solution. This epinephrine dose is a widely used standard dose for CPR in experimental studies using swine models [10,11,12,13].

### 2.2. Experimental Protocol

**Figure 1:**
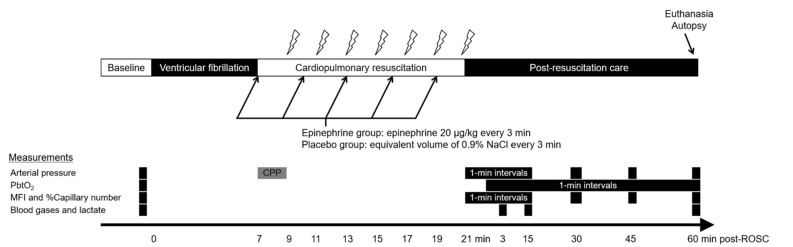
Experimental timeline. After 7 min of untreated ventricular fibrillation, cardiopulmonary resuscitation (CPR) was initiated using a mechanical chest compression device. The lightning marks indicate the onset of a 10-s pause in chest compression for rhythm analysis and a 150-J electric shock, if indicated. After ROSC, the animals were observed for 60 min in a simulated intensive care setting. PbtO_2_ was sampled at baseline and 1-min intervals from 2 min after ROSC. Arterial pressure, MFI, and % capillary number were measured at baseline, 1-min intervals for 15 min after ROSC, and 30, 45, and 60 min after ROSC. Arterial and jugular venous blood gases and lactate levels were measured at baseline and 3, 15, and 60 min after ROSC. CPP was measured for 2 min after the initiation of CPR, as most of the animals (94.7%) achieved ROSC 2–4 min after CPR. PbtO_2_, brain tissue O_2_ tension; MFI, microvascular flow index; % capillary number, percent of counted number of perfused capillaries relative to that at the baseline; CPP, coronary perfusion pressure; ROSC, restoration of spontaneous circulation.

Immediately after baseline measurements were obtained, VF was induced by delivering a 60 Hz and 30 mA alternating current through the pacing catheter placed in the right ventricle. Mechanical ventilation was suspended immediately after VF induction. After 7 min of untreated cardiac arrest (Figure 1), external chest compressions using a pneumatic, piston-driven chest compressor (Life-Stat; Michigan Instruments, Grand Rapids, MI, USA) were started at a rate of 100 compressions/min to a depth of approximately 20% of the anterior-posterior chest diameter. Coincidentally, with the start of chest compression, ventilation was resumed with high-flow O_2_ (15 L/min) at a rate of 10 ventilations/min. The duration of untreated cardiac arrest in the present study was chosen based upon the interval between emergency call and ambulance arrival at scene in the PARAMEDIC2 trial (median 6.7 min and 6.6 min in the epinephrine and placebo groups, respectively) [3]. Either saline placebo or epinephrine solution was administered intravenously every 3 min, beginning immediately after the start of CPR. Defibrillation was attempted with a 150-J biphasic waveform transthoracic shock every 2 min. CPR was continued until ROSC (unassisted pulsatile rhythm with a systolic arterial pressure > 60 mmHg) or for 14 min. The resuscitation efforts were discontinued if ROSC was not attained within 14 min of CPR.

Animals that attained ROSC underwent a 60-min period of intensive care under general anesthesia with sevoflurane. The dose of sevoflurane used during this period was 1% in all animals; no animals exhibited reflex withdrawal at this dose. Immediately after ROSC, mechanical ventilation was resumed with 100% O_2_, with the other ventilator settings as pre-arrest. The ventilatory rate was adjusted to maintain an end-tidal CO_2_ of 40 mmHg after 15 min following ROSC. During this period, normal saline (10 mL/kg) was infused to maintain normovolemia, but no hemodynamic drugs were given as the mean arterial pressure was maintained at higher than 65 mmHg in all animals. None of the animals reached the predetermined humane endpoints for euthanasia (systolic arterial pressure < 60 mmHg, heart rate < 40 beats/min, or seizure) prior to completion of the experimental protocol. At the end of the 60-min period, the animals were humanely euthanized using a rapid bolus of 40 mEq potassium chloride under deep sevoflurane anesthesia (5%). An autopsy was performed on each animal to check for internal organ injuries.

### 2.3. Measurements

Aortic pressure, right atrial pressure, and electrocardiographic data were measured using a hemodynamic monitor (CS/3 CCM; Datex-Ohmeda, Helsinki, Finland) and stored on a personal computer using data collection software (S/5 Collect software; Datex-Ohmeda, Helsinki, Finland). CPP during CPR was calculated as the difference between aortic pressure and right atrial pressure at end-diastole. PbtO_2_ and cerebral MBF were assessed on the right and left parietal cortices (PbtO_2_ on one hemisphere and cerebral MBF on the other hemisphere). The measurement side (right or left) was randomized and counterbalanced. PbtO_2_ was measured with an optical O_2_ sensor (DP-PSt7; PreSens-Precision Sensing GmbH, Regensburg, Germany) placed on the surface of the cerebral cortex. To test the responsiveness of PbtO_2_ to changes in the fraction of inspired O_2_ (FiO_2_), the FiO_2_ was increased from baseline (0.3) directly to 1.0 and remained at 1.0 until the PbtO_2_ level reached a plateau. Then, the FiO_2_ was reset to baseline level. Cerebral hypoxia was defined as a PbtO_2_ below 20 mmHg [6]. To assess the cerebral MBF, an experienced investigator obtained cerebral microcirculation videos using a hand-held microscope (G-Scope G5; Genie Tech, Seoul, Korea) placed over the burr hole. This microscope showed an area of interest of approximately 1800 × 1000 μm^2^ at ×250 magnification. Two investigators blinded to the study group allocations reviewed the obtained videos and measured the microvascular flow index (MFI) for vessels smaller than 20 μm in diameter (capillaries) [14,15]. The image was divided into four quadrants and a score ranging from 0 to 3 (absent flow = 0, intermittent flow = 1, sluggish flow = 2, and normal flow = 3) was determined for each quadrant by consensus of the two investigators. The MFI score was calculated as the averaged value of the four quadrant scores. The same investigators counted the number of perfused capillaries using the method described by Serné et al. [16]. To account for substantial inter-animal variation in the number of perfused capillaries at baseline, the number of perfused capillaries after ROSC was expressed as a percent of the counted number of perfused capillaries relative to that at the baseline (% capillary number). Mean arterial pressure, MFI, and percentage capillary number were sampled at baseline, 1-min intervals for 15 min after ROSC, and at 30, 45, and 60 min after ROSC. PbtO_2_ was sampled at baseline and 1-min intervals from 2 min after ROSC. Arterial and jugular venous blood gases and lactate levels were measured (GEM Premier 3000; Instrumentation Laboratory Company, Lexington, MA, USA) at baseline and 3, 15, and 60 min after ROSC. The following oxygenation and metabolic parameters were calculated from the blood gas and lactate measurements: the difference between jugular venous and brain tissue O_2_ tension (jugular venous PO_2_ [PjvO_2_]—PbtO_2_); arterial O_2_ content ([1.34 × hemoglobin × arterial O_2_ saturation] + [0.0031 × partial pressure of arterial O_2_ {PaO_2_}]); jugular venous O_2_ content ([1.34 × hemoglobin × jugular venous O_2_ saturation] + [0.0031 × partial pressure of jugular venous O_2_ {PjvO_2_}]); the difference between arterial and jugular venous O_2_ content (arterial O_2_ content—jugular venous O_2_ content); cerebral O_2_ extraction fraction ([{arterial O_2_ content—jugular venous O_2_ content}/arterial O_2_ content] × 100); the difference between jugular venous and arterial CO_2_ tension (partial pressure of jugular venous CO_2_ [PjvCO_2_]—partial pressure of arterial CO_2_ [PaCO_2_]); the ratio of the difference between jugular venous and arterial CO_2_ tension to the difference between jugular venous and arterial O_2_ content ([PjvCO_2_—PaCO_2_]/[arterial O_2_ content—jugular venous O_2_ content]); the difference between jugular venous and arterial lactate (jugular venous lactate—arterial lactate); and the lactate oxygen index ([jugular venous lactate—arterial lactate]/[arterial O_2_ content—jugular venous O_2_ content]) [17,18,19,20,21].

### 2.4. Statistical Analysis

The primary outcome of the study was PbtO_2_. The secondary outcomes were MFI, % capillary number, hemodynamic and blood gas parameters (mean arterial pressure, heart rate, PaO_2_, and PaCO_2_), PjvO_2_—PbtO_2_, cerebral O_2_ extraction fraction, arterial and jugular venous lactate, the ratio of the difference between jugular venous and arterial CO_2_ tension to the difference between arterial and jugular venous O_2_ content, the difference between jugular venous and arterial lactate, the lactate oxygen index, CPP during CPR, ROSC rate, and duration of CPR. Data were analyzed using R software version 3.3.3 (R Foundation for Statistical Computing, Vienna, Austria) and T&F program version 3.0 (YooJin BioSoft, Goyang, Korea). Continuous variables were tested for normality using the Shapiro–Wilk and Kolmogorov–Smirnov tests. Normally distributed continuous variables were summarized by their means ± standard deviation and independent two-sample t tests were used to identify differences between groups, while non-normally distributed continuous variables were summarized by their medians and interquartile ranges (IQR); Mann–Whitney U tests were used to identify differences. Paired sample t tests or Wilcoxon signed-rank tests were used for within-group comparisons of continuous variables, depending on the normality of the variables. Categorical variables were compared using Fisher’s exact test. Areas under the curves (AUC) were calculated and summarized as mean ± standard error or median (IQR) for intergroup comparisons of data obtained during and after CPR, except variables that contained both positive and negative values (difference between jugular venous and brain tissue O_2_ tension, difference between jugular venous and arterial CO_2_ tension, ratio of difference between jugular venous and arterial CO_2_ tension to difference between arterial and jugular venous O_2_ content, difference between jugular venous and arterial lactate, and lactate oxygen index). The AUCs for data obtained during the 60-min post-ROSC period were separately calculated for the data from the first 10-min period and those from the subsequent 50-min period (or the first 15-min period and those during the subsequent 45-min period for blood gas- and lactate-derived variables), as well as for the data during the entire 60-min post-ROSC period. Linear mixed effect models were generated to analyze group effects and/or time effects on the variables that contained both positive and negative values. The linear mixed models contained baseline measurement, time, treatment group, and interaction of time and treatment group as fixed effects with random intercept. Bonferroni corrections were used to correct for multiple comparisons. The sample size for this study was calculated based on the PbtO_2_ data from a pilot study, in which the AUC of PbtO_2_ during the subsequent 50-min period was 2665 ± 644 and 1421 ± 563 mmHg·min in the placebo and epinephrine groups, respectively. We calculated that five animals would be required per group to reach an α of 0.05 and a power of 80%. Assuming an ROSC rate of 40% in the placebo group, we included 12 animals per group. A two-tailed *p* value of <0.05 was considered statistically significant.

## 3. Results

Of the 24 animals included in the present study, one animal in the epinephrine group was excluded from the analysis due to CPR-related hemothorax, leaving 12 animals in the placebo group and 11 in the epinephrine group. There were no between-group differences in the baseline measurements (Table 1). At baseline, increasing the FiO_2_ from 0.3 to 1.0 resulted in a marked increase in PbtO_2_, from 32.8 ± 6.1 mmHg to 76.3 ± 17.4 mmHg (*p* < 0.001). The epinephrine group received 500 (478–804) μg of epinephrine during CPR (number of epinephrine doses, 1 [1–1.5]). The AUC of CPP during CPR was significantly higher in the epinephrine group than in the placebo group (17.2 ± 2.7 mmHg·min versus 8.9 ± 1.7 mmHg·min, *p* = 0.015). The ROSC rate was comparable between the placebo and epinephrine groups (8 [66.7%] versus 11 [100%], *p* = 0.093), but the epinephrine group required a shorter duration of CPR than the placebo group (2 [2–4] min versus 4 [3–12] min, *p* = 0.017). All resuscitated animals in both groups were hemodynamically stabilized without vasopressor support, and survived the 60-min post-ROSC period.

**Figure 2:**
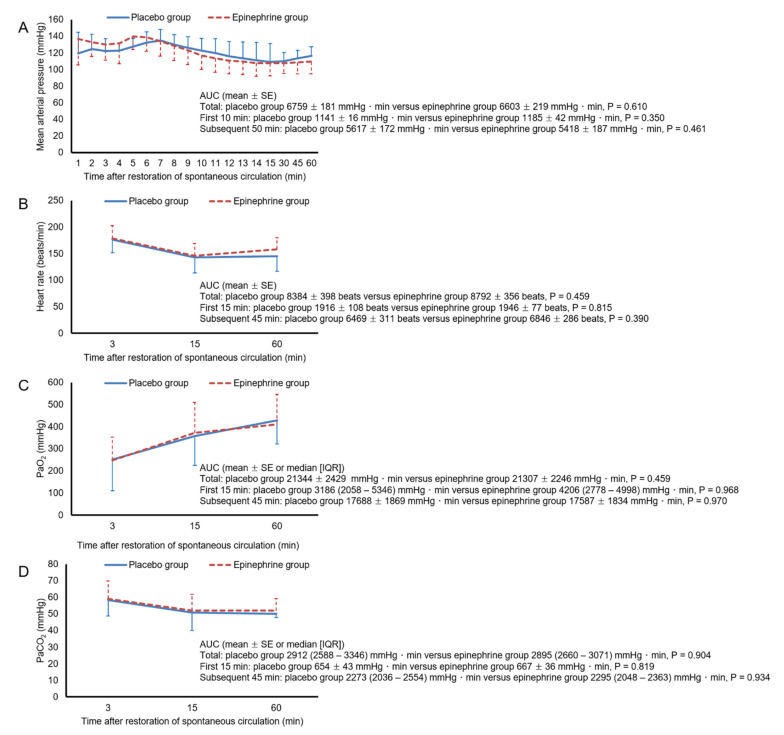
Mean arterial pressure (**A**), heart rate (**B**), PaO_2_ (**C**), and PaCO_2_ (**D**) after the restoration of spontaneous circulation. Error bars represent the standard deviation. AUC, area under the curve; SE, standard error; IQR, interquartile range; PaO_2_, partial pressure of arterial O_2_; PaCO_2_, partial pressure of arterial CO_2_.

**Figure 3:**
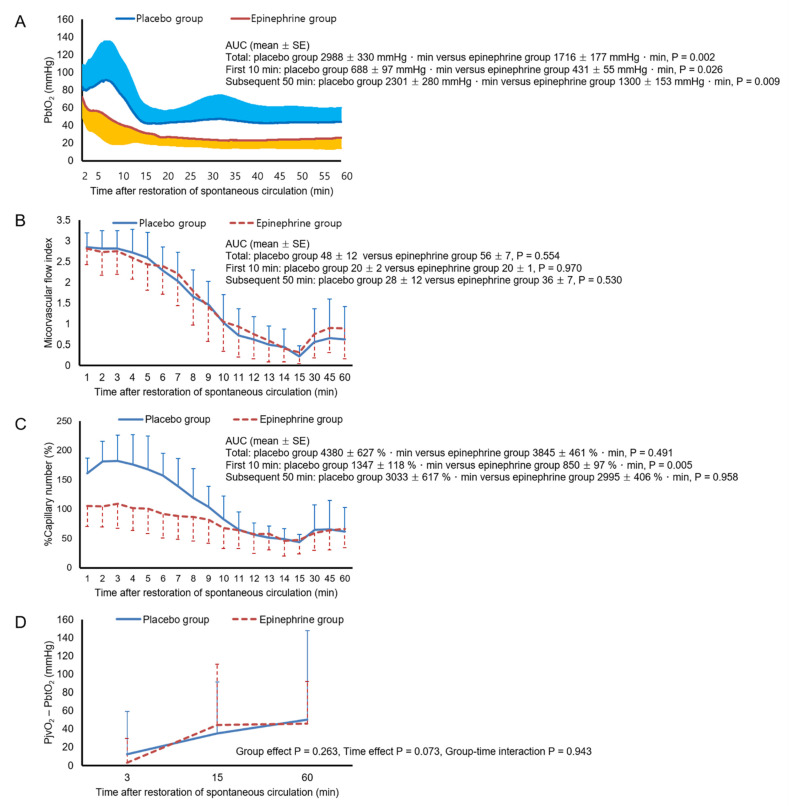
Brain tissue O_2_ tension (PbtO_2_, **A**), microvascular flow index (**B**), % capillary number (**C**), and partial pressure of jugular venous O_2_ (PjvO_2_)—PbtO_2_ (**D**) after the restoration of spontaneous circulation. Error bars represent the standard deviation. AUC, area under the curves; SE, standard error; percentage capillary number, percent of counted number of perfused capillaries relative to that at the baseline.

**Figure 4:**
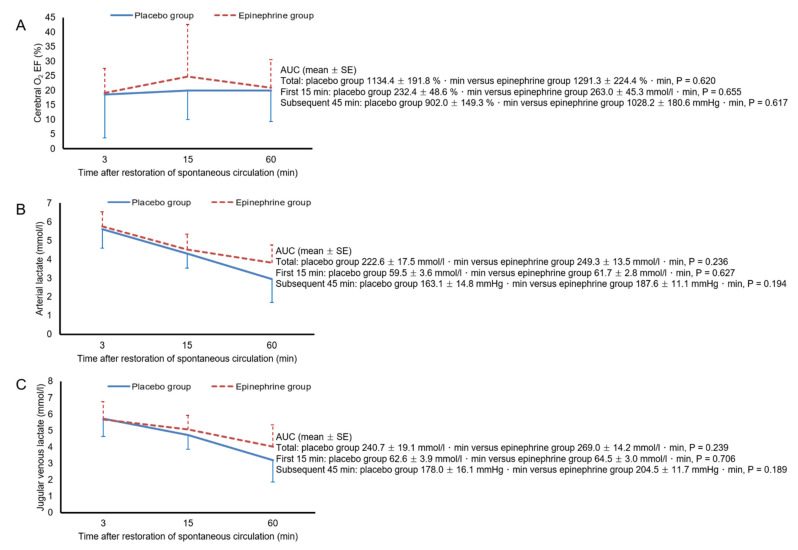
Cerebral O_2_ extraction fraction (EF, **A**), arterial lactate (**B**), and jugular venous lactate (**C**) levels after the restoration of spontaneous circulation. Error bars represent the standard deviation. AUC, area under the curves; SE, standard error.

**Figure 5:**
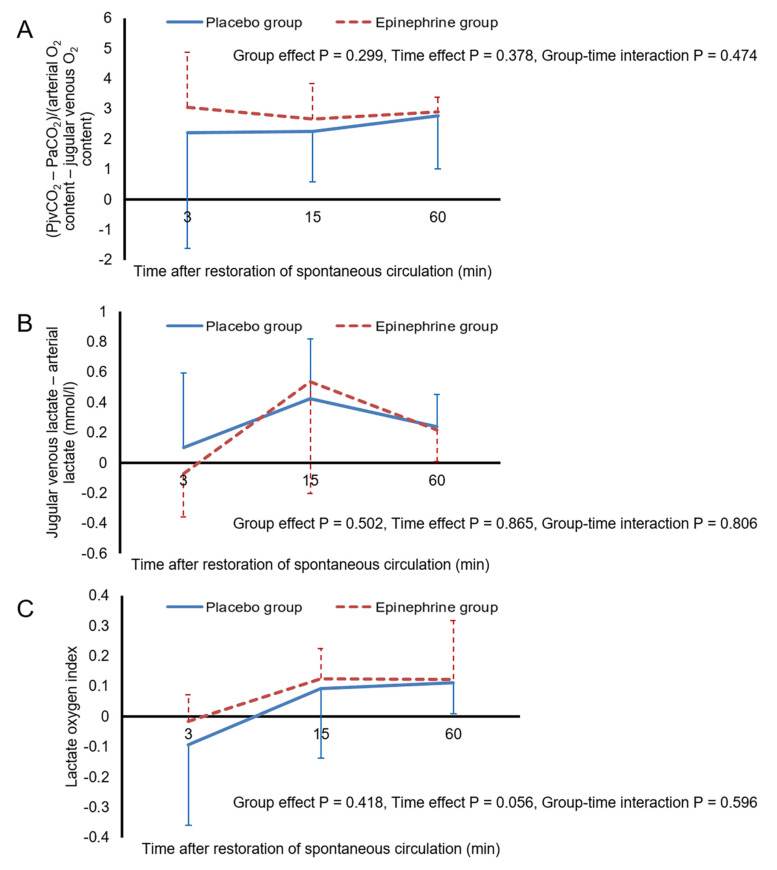
(PjvCO_2_—PaCO_2_)/(arterial O_2_ content—jugular venous O_2_ content) (**A**), jugular venous lactate—arterial lactate (**B**), and lactate oxygen index (**C**) after the restoration of spontaneous circulation. Error bars represent standard deviation. PjvCO_2_, partial pressure of jugular venous CO_2_; PaCO_2_, partial pressure of arterial CO_2_.

Figure 2 shows the mean arterial pressure, heart rate, PaO_2_, and PaCO_2_ values during the 60-min post-ROSC period. There were no significant differences in the AUCs for mean arterial pressure, heart rate, PaO_2_, or PaCO_2_ between the two groups, during either the first 10-min period or the subsequent 50-min period. In both groups, mean arterial pressure remained above 80 mmHg throughout the 60-min post-ROSC period. PaO_2_ increased and PaCO_2_ decreased progressively over time, but no animals had hypoxemia or hypocapnia during this period. Figure 3 shows cerebral measurement data during the 60-min post-ROSC period. Throughout this period, PbtO_2_ remained lower in the epinephrine group than in the placebo group. The AUC for PbtO_2_ was significantly smaller in the epinephrine group than in the placebo group for both the first 10-min period and the subsequent 50-min period (*p* = 0.002 and 0.009, respectively). In the placebo group, two animals experienced cerebral hypoxia, for 5 min and 9 min, as did five animals in the epinephrine group, for 40 (39–43) min. However, neither the number of animals that experienced cerebral hypoxia nor the duration of exposure to cerebral hypoxia differed significantly between the two groups. The AUC for MFI did not differ between the two groups in either the first 10-min period or the subsequent 50-min period. The AUC for percentage capillary number during the first 10-min period was significantly smaller in the epinephrine group (*p* = 0.005), but that for the subsequent 50-min period was comparable between the two groups. Neither group effect nor group–time interaction was found with respect to the PjvO_2_—PbtO_2_. As shown in Figure 4, there were no significant differences in the AUCs for cerebral O_2_ extraction fraction, arterial lactate, or jugular venous lactate between the two groups in either the initial 15-min period or the subsequent 45-min period. Figure 5 shows the ratio of the difference between jugular venous and arterial CO_2_ tension to the difference between arterial and jugular venous O_2_ content, the difference between jugular venous and arterial lactate, and the lactate oxygen index. Neither significant group effects nor group-time interactions were found with respect to these metabolic parameters.

## 4. Discussion

Hypoxic brain injury is a significant determinant of neurological outcome after cardiac arrest. To prevent secondary hypoxic brain injury after ROSC and thus improve neurologically favorable survival, it is critically important to ensure sufficient cerebral oxygenation during the post-resuscitation period. Given that epinephrine is still the most commonly used medication in cardiac arrest, a better delineation of the effect of epinephrine on cerebral oxygenation is required to fill the knowledge gap regarding the effect of epinephrine on neurologic outcome after cardiac arrest. In the present study, in which we investigated the effects of epinephrine administered during CPR on cerebral oxygenation in a swine model, epinephrine led to significantly lower PbtO_2_ for the first 10 min as well as for the subsequent 50 min. The persistent reduction of PbtO_2_ in the epinephrine group led to a higher incidence and longer duration of cerebral hypoxia in the epinephrine group, although these differences did not reach statistical significance, probably due to the small sample size.

Although limited data are available on the relationship between PbtO_2_ and neurologic injury after cardiac arrest [22,23], a number of studies have suggested that the depth and duration of low PbtO_2_ values correlate with unfavorable outcomes in patients with severe traumatic brain injury [24,25,26,27]. Our findings, together with those of these studies [24,25,26,27], indicate a plausible link between PbtO_2_ and neurologic outcome among the survivors of the epinephrine group in the PARAMEDIC2 trial. The epinephrine-induced, persistent reduction of PbtO_2_ in the present study, in contrast to the rapid reversal of epinephrine-induced reduction of PbtO_2_ observed in the study by Ristagno et al. [5], might be due to the prolonged duration before treatment of cardiac arrest in the present study. No studies, to the best of our knowledge, have evaluated the effects of the duration of untreated cardiac arrest on the epinephrine-induced reduction of PbtO_2_, but several experimental studies using a model with relatively prolonged duration of untreated cardiac arrest reported findings suggestive of sustained reduction in PbtO_2_ after ROSC [9,23]. In a study in which rats underwent 9 min or 12 min of asphyxial cardiac arrest followed by CPR including epinephrine administration [9], cortical PbtO_2_ was similar to baseline until 15 min after ROSC but was lower than baseline from 30 min to 120 min after ROSC. The reduction in PbtO_2_ was greater after 12 min asphyxial cardiac arrest than after 9 min. Several studies in comatose cardiac arrest patients have demonstrated cerebral hypoxia and ischemic neurochemical changes after several hours following ROSC [21,28,29]. We believe that our protocol, compared with those from models with a shorter untreated cardiac arrest duration, provided a better facsimile of the pathophysiological changes in comatose cardiac arrest patients.

The lower PbtO_2_ during the first 10-min period in our epinephrine group may be attributable to attenuation of the initial hyperemia after ROSC in the epinephrine group. Several studies have demonstrated the occurrence of transient cerebral hyperemia immediately following ROSC, especially after prolonged cardiac arrest [7,23,30]. Consistent with these studies [7,23,30], our data showed that the number of perfused capillaries was higher during the first 10-min period than at baseline for both groups, but this was significantly less prominent in the epinephrine group. In contrast to the percentage capillary number, the MFI did not differ between the two groups during this period. This might have resulted from the inability of the MFI score to differentiate between normal flow and hyperemia. Several studies suggest that post-ischemic hyperemia is detrimental, as it induces vascular injury and aggravates neurologic injury [31,32]. However, the impact of the epinephrine-induced attenuation of initial hyperemia on neurologic injury remains to be elucidated.

In the present study, none of the hemodynamic, blood gas, or cerebral microcirculation parameters explained the lower PbtO_2_ in the epinephrine group during the 50-min period: there were no significant between-group differences with regard to MAP, PaCO_2_, PaO_2_, MFI, or percentage capillary number, all of which are known to be determinants of cerebral O_2_ delivery [30,33,34,35]. Among these, the lack of significant between-group differences in MFI and percentage capillary number contrasted with the findings of the study by Ristagno et al., in which epinephrine-induced reduction of PbtO_2_ was accompanied by decreases in MFI and the number of perfused capillaries [5]. One possible explanation for these findings is that the MFI failed to capture a genuine between-group difference in cerebral microvascular flow; MFI is a fairly coarse measure, with only four categories. In a study that analyzed 100 sublingual microcirculation videos obtained from 25 patients with septic shock [36], MFI explained only 36% of the variance in the velocity of red blood cells in the videos as measured using specific analysis software. Further study using tools that enable finer measurement of cerebral microvascular flow is needed to evaluate this possibility.

PbtO_2_ is determined not only by cerebral MBF but also by other factors, including diffusion limitation in cerebral O_2_ delivery and cerebral O_2_ consumption. The difference in PbtO_2_ between the two groups might be attributable to differences in other determinants of PbtO_2_. In a study that investigated the relationship between the determinants of cerebral O_2_ delivery and PbtO_2_ during the 60-min post-ROSC period in a swine cardiac arrest model [30], MFI and percentage capillary number could explain only a small proportion of the PbtO_2_ variance (semi-partial R^2^ = 0.143 [95% confidence interval, 0.062–0.247] and 0.113 [95% confidence interval, 0.040–0.212], respectively), suggesting the presence of factors whose influence on PbtO_2_ was greater than that of cerebral MBF.

Epinephrine induces an increase in cerebral O_2_ consumption [37,38]. The lower PbtO_2_ in our epinephrine group could have resulted from increased cerebral O_2_ consumption. However, there were no significant intergroup differences in any of the cerebral metabolic parameters, including the ratio of the difference between jugular venous and arterial CO_2_ tension to the difference between arterial and jugular venous O_2_ content, the difference between jugular venous and arterial lactate, and the lactate oxygen index. Jugular venous blood is representative of mixed cerebral blood. The decreased cerebral cortical microcirculation after ROSC found in the present study might have caused shunting of blood flow away from any cerebral region with impaired microcirculation and toward those with intact microcirculation, thus limiting the ability of jugular venous blood gases and lactate to reflect metabolic changes in cerebral regions with impaired microcirculation. Further study is required to investigate the effect of epinephrine on cerebral O_2_ consumption and metabolism in cerebral regions with impaired microcirculation. Similarly, although PjvO_2_—PbtO_2_, which has been used for assessment of the degree of diffusion limitation in cerebral O_2_ delivery [21,22], did not differ between the two groups, this parameter might not be reliable in assessing the degree of diffusion limitation. In a study that included 13 patients with traumatic brain injury, Menon et al. found an increased gradient between cerebral venous PO_2_ near the PbtO_2_ sensor, as estimated using ^15^O_2_ positron emission tomography, and PbtO_2_ in the tissue region with cerebral hypoxia [39]; they suggested diffusion limitation as a mechanism of cerebral hypoxia. Although cardiac arrest, unlike traumatic brain injury, produces global injury to the brain, PbtO_2_ varies markedly depending on the area measured [40]. Given this marked regional variability in cerebral oxygenation, PjvO_2_ might not have accurately represented cerebral venous PO_2_ near the PbtO_2_ sensor in the present study.

In the present study, epinephrine led to significantly higher CPP during CPR and hastened ROSC, consistent with a number of studies reporting the benefits of epinephrine in attaining ROSC [41,42]. However, this was accompanied by a significant and persistent reduction in PbtO_2_ after ROSC. These results are consistent with those of clinical studies suggesting increased ROSC rates after epinephrine, but at the cost of poor neurological outcome [2,3]. Several studies have indicated that cerebral hypoxia is treatable [23,27]. Elmer et al. compared PbtO_2_-guided care, which included titration of MAP, FiO_2_, and positive end-expiratory pressure to maintain a PbtO_2_ ≥ 20 mmHg, with standard care in a swine model of opioid overdose cardiac arrest [23], and reported that PbtO_2_-guided care significantly reduced exposure to cerebral hypoxia compared with standard care. PbtO_2_ monitoring and therapies to reduce exposure to cerebral hypoxia may enable the benefits of epinephrine in achieving ROSC to be accompanied by improved neurologically favorable survival.

This study has several limitations. Firstly, it was conducted on young, healthy, and anesthetized animals. Secondly, unlike CPR in a typical out-of-hospital cardiac arrest resuscitation setting, basic life support was not given, and the first dose of epinephrine was provided immediately after the start of CPR. Accordingly, the first dose of epinephrine was provided after much briefer period of time in the present study (7 min) than in the PARAMEDIC2 trial (21 min) [3]. These differences may limit the extrapolation of our findings. Thirdly, unlike a typical clinical setting, where FiO_2_ is reduced during the post-ROSC period to avoid hyperoxemia, FiO_2_ was maintained at 1.0 throughout the 60-min post-ROSC period to allow the measurement of changes in PbtO_2_ over time at a constant FiO_2_. Fourthly, cerebral measurements including MFI, percentage capillary number, and PbtO_2_ were highly focal, and thus might not reflect changes across the whole brain. Finally, we could not explain the mechanism underlying the epinephrine-induced reduction of PbtO_2_ during the subsequent 50-min period, warranting further research.

## 5. Conclusions

In conclusion, epinephrine administered during CPR reduced PbtO_2_ beyond the first 10 min following ROSC in a swine model with a clinically relevant duration of untreated cardiac arrest. Our results provide a plausible explanation for the worse neurological outcome among the survivors of the epinephrine group in the PARAMEDIC2 trial compared to that in the placebo group.

## Figures and Tables

**Table 1:** Baseline measurements.

Variable	Placebo Group (*n* = 12)	Epinephrine Group (*n* = 11)	*p* Value ^1^
Systolic arterial pressure (mmHg)	127 ± 14	127 ± 17	0.988
Diastolic arterial pressure (mmHg)	87 ± 14	84 ± 16	0.607
Mean arterial pressure (mmHg)	104 ± 13	103 ± 15	0.799
Systolic right atrial pressure (mmHg)	10 ± 2	9 ± 2	0.385
Diastolic right atrial pressure (mmHg)	6 (4–7)	4 (3–7)	0.063
Mean right atrial pressure (mmHg)	8 ± 2	7 ± 2	0.167
Heart rate (beats/min)	92 ± 20	87 ± 13	0.506
End-tidal CO_2_ (mmHg)	39 ± 3	38 ± 2	0.480
Rectal temperature (°C)	37.2 ± 0.8	37.4 ± 0.8	0.532
Arterial pH	7.501 ± 0.040	7.523 ± 0.024	0.133
PaCO_2_ (mmHg)	42.7 ± 3.7	40.9 ± 3.0	0.223
PaO_2_ (mmHg)	172.5 ± 39.0	171.8 ± 27.3	0.962
SaO_2_ (%)	100 (99–100)	100 (100–100)	0.247
Arterial lactate (mmol/L)	0.97 ± 0.24	1.01 ± 0.27	0.693
Jugular venous pH	7.424 ± 0.068	7.452 ± 0.031	0.230
PjvCO_2_ (mmHg)	53.3 ± 9.3	50.0 ± 5.0	0.293
PjvO_2_ (mmHg)	48.4 ± 23.0	46.5 ± 14.3	0.811
SjvO_2_ (%)	74 ± 21	80 ± 10	0.377
Jugular venous lactate (mmol/L)	1.36 ± 0.51	1.38 ± 0.64	0.923
Cerebral O_2_ extraction fraction (%)	27.7 ± 20.7	22.0 ± 10.3	0.405
(PjvCO_2—_PaCO_2_)/(arterial O_2_ content—jugular venous O_2_ content)	3.33 ± 0.88	3.73 ± 1.47	0.433
Jugular venous lactate—arterial lactate (mmol/L)	0.25 (0.12–0.6)	0.3 (0.2–0.4)	0.950
Lactate oxygen index	0.11 (0.08–0.16)	0.12 (0.09–0.17)	0.449
PbtO_2_ at FiO_2_ 0.3 (mmHg)	31.6 ± 5.6	34.1 ± 6.7	0.342
PbtO_2_ at FiO_2_ 1.0 (mmHg)	74.3 ± 18.6	78.5 ± 16.5	0.567
Microvascular flow index ^2^	3	3	NA
Number of perfused capillaries	11 ± 3	13 ± 3	0.190
PjvO_2_—PbtO_2_ (mmHg)	16.8 ± 24.1	12.3 ± 16.8	0.616

Data are the means ± standard deviation or medians with interquartile ranges. ^1^ *p* values were calculated using independent two-sample *t* tests or Mann–Whitney U tests. ^2^ Microvascular flow index at baseline was 3 in all animals. PaCO_2_, partial pressure of arterial CO_2_; PaO_2_, partial pressure of arterial O_2_; SaO_2_, arterial O_2_ saturation; PjvCO_2_, partial pressure of jugular venous CO_2_; PjvO_2_, partial pressure of jugular venous O_2_; SjvO_2_, jugular venous O_2_ saturation; PbtO_2_, brain tissue O_2_ tension; FiO_2_, fraction of inspired O_2_, NA, not applicable.

## Data Availability

The data presented in this study are available in Supplementary Material.

## Supplementary Materials

The following is available online at https://www.mdpi.com/article/10.3390/ijerph18115896/s1, File S1: data.
